# Comparing the Efficacies of Telemedicine and Standard Prenatal Care on Blood Glucose Control in Women With Gestational Diabetes Mellitus: Randomized Controlled Trial

**DOI:** 10.2196/22881

**Published:** 2021-05-25

**Authors:** Ying Tian, Suhan Zhang, Feiling Huang, Liangkun Ma

**Affiliations:** 1 Department of Obstetrics and Gynecology, National Clinical Research Center for Obstetric & Gynecologic Diseases Peking Union Medical College Hospital Chinese Academy of Medical Sciences & Peking Union Medical College Beijing China

**Keywords:** gestational diabetes mellitus, blood glucose, mhealth, health education, lifestyle management

## Abstract

**Background:**

Gestational diabetes mellitus (GDM) can usually be well controlled by health education and lifestyle management, resulting in better pregnancy outcomes. However, standard clinical prenatal care, which consists of clinic visits every 2 weeks, may not provide sufficient management for women with GDM. Telemedicine demonstrates a potential to fill this gap.

**Objective:**

The objective of this study was to investigate whether health education and lifestyle management delivered through a WeChat group chat was more effective in controlling blood glucose (BG) than standard clinical prenatal care among women with GDM.

**Methods:**

In this multicenter randomized controlled trial, women with GDM diagnosed by an oral glucose tolerance test between 23 and 30 (+6) gestational weeks were randomized to a WeChat group chat–based BG management group or a routine clinical prenatal care group. The primary outcome was the change in the glycemic qualification rate during the follow-up period in both groups. The secondary outcomes were pregnancy outcomes.

**Results:**

A total of 309 women with GDM participated in the trial, with 162 women randomized to the control group and 147 to the intervention group. No significant differences in baseline characteristics were found between the control and intervention groups. Participants were further divided into 4 groups according to gestational weeks at enrollment for further analysis. The glycemic qualification rate of the intervention group was higher than that of the control group at nearly all time points in Groups 1 to 3, among which 3 time points reached statistical significance: Group 1 at T3 (54.8% vs 83.3%) and Group 2 at T3 (62.5% vs 80.0%) and T7 (75.0% vs 100%). The glycemic qualification rate gradually increased as gestational weeks progressed in both groups, regardless of the intervention method. None of the pregnancy outcomes measured, including delivery mode, premature rupture of the membranes, preterm birth, infant's birth weight, and postpartum hemorrhage, were significantly different between the control and intervention groups.

**Conclusions:**

This multicenter randomized controlled trial that assessed women with noninsulin-dependent GDM demonstrated that additional instant messaging platforms, such as WeChat, used for health education and lifestyle intervention in China tend to be more effective for BG control than standard clinical prenatal care alone.

**Trial Registration:**

ClinicalTrials.gov NCT03748576; https://clinicaltrials.gov/ct2/show/NCT03748576

## Introduction

Gestational diabetes mellitus (GDM) has been defined as carbohydrate intolerance resulting in hyperglycemia of variable severity with an onset or first recognition during pregnancy that resolves after delivery [1]. Although this condition of carbohydrate intolerance is maintained only during the peripartum period, the potential risks are not limited to pregnancy outcomes. Short-term impacts include a higher risk of developing gestational hypertension and preeclampsia; an increased risk of preterm birth, cesarean section, perineal trauma, and postpartum hemorrhage for mothers [2,3]; and an increased risk of macrosomia, large for gestational age status, birth trauma, and neonatal hypoglycemia for infants [4]. Potential long-term effects include but are not limited to a risk of developing type 2 diabetes within 5 to 10 years, higher risks of developing cardiovascular disease for mothers [5], and higher rates of obesity, late-onset diabetes, and cardiovascular disease in adulthood for infants [6-8]. Certain ethnic groups, such as Asians, have a higher risk of GDM [9]. The aim of GDM treatment and patient education is to keep glucose levels within the recommended glycemic reference range to prevent maternal hyper- or hypoglycemia [10,11]. Lifestyle modifications, including diet consultation and physical exercise, are considered the first-line intervention and may sufﬁce for most patients [10]. Women with GDM who are on insulin therapy receive more attention from doctors and researchers, while women with GDM with mild dysglycemia who control their condition with diet alone receive much less attention, although this group may account for the majority of patients with GDM [12].

Patient education—including improving GDM-related knowledge and clarifying the importance of self-management, and multidisciplinary consultation—including blood glucose (BG) monitoring, lifestyle modifications, and drug therapy—are necessary for women with GDM after receiving a GDM diagnosis. Additionally, there are some cell phone applications and WeChat Official Accounts that are specifically for women to use during pregnancy to improve disease knowledge and monitor BG and body weight. Regardless of access to medical advice and self-management, managing and educating women with GDM is not consistent, and the adherence of patients is unsupervised in most cases. These factors may lead to consequences, such as reducing GDM management efficiency and causing poor pregnancy outcomes [13]. It was reported that good adherence of patients with GDM to clinical recommendations was observed, especially in relation to BG monitoring, but nutritional guidelines were less likely to be followed [14]. Given the limited intervention time window, the importance of continuous patient education and self-management supervision during the periods between antenatal care visits should be strongly emphasized.

The rapid development of the internet and smartphones has caused telemedicine to be more convenient. For women with GDM, online management can potentially improve the likelihood of following medical advice and keep patients with GDM alert to the harm of poor BG control, further achieving continuous disease management. Research has demonstrated the acceptability and feasibility of mobile medical management of women with GDM through social software platforms, instant messaging applications, web pages, and regular email alerts or telephone visits. Some of the related studies did show a slight correlation between mobile health applications and BG levels, as well as better efficacy and greater demand for more attention, communication, and management from a majority of women with GDM [15-17]. However, to date, telemedicine approaches have failed to show significant advantages over standard prenatal care, especially in terms of clinical outcomes, when used for GDM. Possibly because of the limited sample sizes and immature intervention paths, it is difficult to identify whether a relationship between technology use and clinical outcomes exists [15-23]. A large-sample randomized controlled study is needed to further explore the application value of mobile medicine regarding BG control, health management, and clinical outcomes.

WeChat, an instant messaging platform with more than 1.13 billion monthly global active users, is widely used not only for message delivery but also gradually in the medical field, including but not limited to online consultation, patient group management, and live patient education. The potential of WeChat for telemedicine applications cannot be ignored. We designed a WeChat group chat–based GDM management program to conduct health education and lifestyle management online to facilitate continuous patient education and self-management during the periods between prenatal care visits. The patient management team included obstetricians, nutritionists, nurses, and health managers at a minimum, and psychologists and sports medicine doctors were included if needed. Based on our clinical experience and patients’ responses, we hypothesized that health education and lifestyle management offered by medical experts on WeChat would result in better control of BG and better pregnancy outcomes. This randomized controlled trial was conducted to verify this hypothesis by comparing the efficacy of health education and lifestyle management conducted through the WeChat group chat on BG control with that of standard clinical prenatal care in women with GDM.

## Methods

### Study Design

This was a multicenter randomized controlled trial initiated by the Chinese Academy of Medical Sciences and Peking Union Medical College Hospital and conducted in 8 prenatal care institutions between June 2019 and December 2019. The trial received ethical approval from the Peking Union Medical College Hospital's Medical Ethics Review Committee (ID: JS-1012). Written informed consent was obtained from each participant. All researchers involved had been trained uniformly before the trial started, and all centers received site instruction when they enrolled the first several participants.

### Participants

The participant inclusion criteria were as follows: (1) aged between 18 and 45 years; (2) singleton pregnancy of fewer than 31 gestational weeks; (3) GDM diagnosis according to the 75 g oral glucose tolerance test (OGTT) [24] and no requirement for insulin treatment according to multidisciplinary consultation; (4) ability to use a smartphone for chatting, reading, and writing basic Chinese; and (5) voluntary participation in research. Pregnant women with a diagnosed chronic disease and other pregnancy complications except GDM, as well as those who had recent trauma or treatment with glucocorticoids, were excluded.

Eligible patients according to assessment by clinic doctors based on the abovementioned criteria received detailed information about the project from the research team. Interested patients completed questionnaires after providing signed informed consent and were randomized to a study group. During the study, all subjects could withdraw at any time without providing a reason. Participants were informed that they would receive postpartum counseling for free as a reward for participating in the study.

### Sample Size

The sample size for this superiority trial was calculated as Inline graphic 1, where P_C_ was the BG control rate in the control group and P_T_ was the expected BG control rate in the test group (referring to clinical data). The absence of published estimates of glycemic control rates made sample size calculations more challenging. We decided to assume that the control rate in the control group was approximately 75%, based on our clinical experience, and that the control rate in the experimental group would be 15% higher than that in the control group. Based on these assumptions, we calculated the sample size to be 104 cases in each group at the significance level of .05 (bilateral) and 80% assurance. Considering a dropout rate of up to 20%, the final sample size was determined to be 125 women in each group.

### Allocation

Participants were randomly allocated to two groups: intervention (WeChat group management) or control (standard clinical prenatal care). A random number table was used to generate the grouping envelope. Participants were grouped according to the random number in the grouping envelope, ensuring randomized allocation. After the results of the grouping were announced, the subjects were not permitted to switch groups.

### Intervention and Control

#### Standard Clinical Prenatal Care (Control Group)

All women diagnosed with GDM were asked to attend one GDM management lesson in a maternity school organized by prenatal care institutions, which were established according to the standards of the Beijing Municipal Health Commission. Participants were taught basic information about GDM and how to do self-management, including how to conduct BG monitoring, what the target BG values are, and how to keep a lifestyle diary. The routine prenatal care appointments were changed to once every 2 weeks when GDM was diagnosed. Doctors generally asked women with GDM to record 5 BG values daily—fasting and pre-sleep BG, and 2-hour postprandial BG (postbreakfast, postlunch, and postdinner BG)—and schedule clinic visits with at least 3 days between visits. At clinic visits, doctors checked the details of patients' lifestyle diaries, including daily diet, exercise, weight, BG, and blood pressure, and lifestyle guidance was provided based on their records. The guidance involved simple principles of a healthy lifestyle such as increasing food diversity, consuming high-fiber cereals, avoiding eating outside, and taking more exercise for weight control. If patients failed to present their diaries, doctors asked them to return with the records on the following week. If the BG could not be controlled after general lifestyle guidance, drug-based interventions were considered after multidisciplinary consultation.

#### WeChat Group Management (Intervention Group)

Patients in the intervention group received WeChat group management in addition to maternity school and standard clinical prenatal care. In the WeChat group chat, participants received management on a weekly basis. In particular, every Monday, researchers would issue a briefing to encourage patients to take an active part in the control of their GDM and a task card to pinpoint the basic requirements, including diet advice, examples of meals from other group members, and exercise rules. Patients performed self-management according to the basic criteria provided for their actual situation and shared photos of their meals and snacks, daily exercise, and experience regarding BG control. Researchers would give individualized guidance for self-management or use a group member’s situation as an example for others. In this way, participants could learn not only from their personalized guidance but also from the experiences of other group members. On weekends, the researchers prepared lessons and articles for group members to learn different aspects of pregnancy and GDM, including rudimentary knowledge, disease management, psychology, and past cases. We encouraged the sharing of learning experiences and notes in the form of peer interactions and support groups. If there were any questions regarding the project, pregnancy, or GDM, patients could seek answers from the group chat. This weekly management continued until delivery.

### Follow-Up Plan

Participants visited the obstetric clinic for prenatal care and follow-up every 2 weeks beginning at the time of enrollment, and each 2-week period was referred to as a “T.” We asked the participants in both groups to record their fasting, postbreakfast, postlunch, postdinner, and pre-sleep BG values for 6 days in 1 T. In other words, within a T, we obtained 30 monitored values. For example, if someone enrolled at 24 gestational weeks and delivered at 40 gestational weeks, she would provide 8 follow-up records; 24 gestational weeks would be recorded as T0, and the follow-up records would be recorded from T1 to T8. Conversely, if a participant enrolled at 30 gestational weeks and delivered at 38 gestational weeks, she would have only 4 follow-up records, with T0 as the enrollment visit and T1 to T4 as the follow-up period.

### Statistical Analysis

The primary outcome in the study was the glycemic qualification rate. The glycemic qualification rate was calculated as the number of BG levels within the control range/30 × 100%. The BG control ranges were fasting BG (fasting and pre-sleep BG) <95 mg/dL (5.3 mmol/L) and 2-hour postprandial BG (postbreakfast, postlunch, and postdinner BG) <120 mg/dL (6.7 mmol/L) [25]. Secondary outcomes including delivery mode, premature rupture of the membranes, preterm birth, infant's birth weight, and postpartum hemorrhage were compared between the study groups.

Data analysis was carried out using SAS software, version 9.4 (SAS Institute Inc). The descriptive statistics of continuous data are expressed as mean (SD). For comparisons between the groups, the *t* test was used if both groups fulfilled the criteria of the test of normality and homogeneity of variance; otherwise, the nonparameter Wilcoxon rank sum test was used. Descriptive statistics are expressed as n (%), and differences in categorical variables were assessed using the chi-square test. Boxplots were used to illustrate changes in BG during pregnancy. Differences in outcomes between the 2 groups were compared using chi-square analysis. *P*<0.05 was considered statistically significant.

## Results

### Participant Characteristics

**Figure 1:**
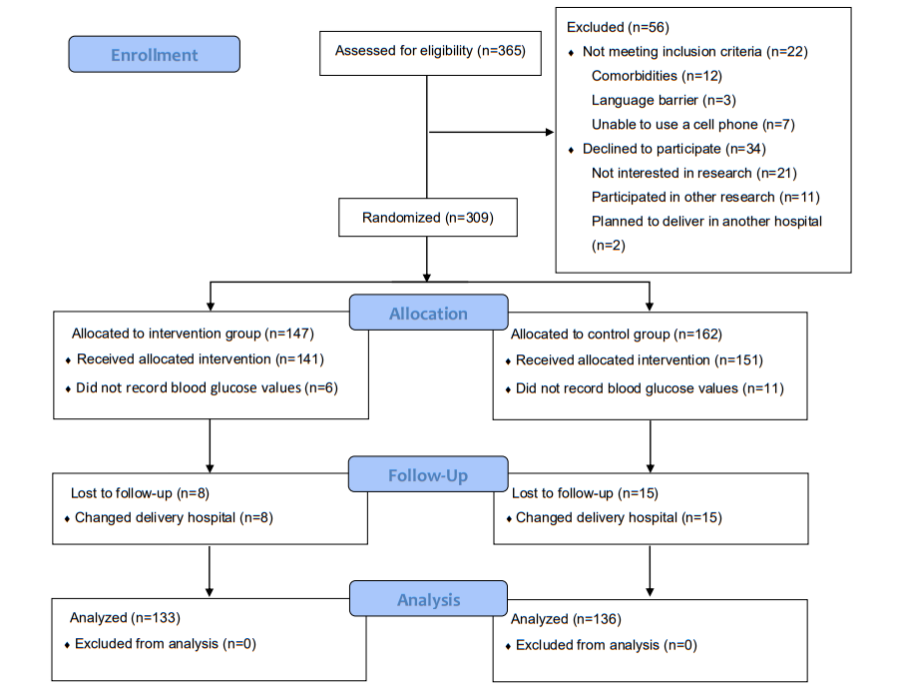
Study flowchart.

As shown in Figure 1, 365 women were screened when they came to the prenatal clinic to check OGTT results; 56 of them were excluded because they did not meet the inclusion criteria (n=22) or they declined to participate (n=34). Of 309 women with GDM who met the recruitment criteria and signed informed consent forms, 162 were randomized to the control group and 147 were randomized to the intervention group. Eleven women in the control group and 6 women in the intervention group did not record any BG values. Fifteen women in the control group and 8 women in the intervention group changed their delivery hospital; thus, delivery records were not available. None of the participants switched to insulin therapy during the program because of persistently severe glucose abnormality or poor pregnancy outcome. Data from 136 women in the control group and 133 in the intervention group were included in the analysis.

No significant differences were found between the control and intervention groups at baseline (Table 1; and Multimedia Appendix 1, Tables 1-4). Of the 269 participants in the analysis, 56 (20.8%) women were over age 35 years, 71 (26.4%) were overweight, and 37 (13.8%) were obese. The OGTT results at enrollment were as follows: fasting BG 4.98 (SD 0.78) mmol/L, 1-hour BG 10.20 (SD 1.84) mmol/L, and 2-hour BG 8.89 (SD 1.69) mmol/L. Participants could be further divided into 4 groups according to gestational weeks at enrollment: Group 1, 66 women who enrolled between 23 and 24 (+6) gestational weeks; Group 2, 113 women who enrolled between 25 and 26 (+6) gestational weeks; Group 3, 66 women who enrolled between 27 and 28 (+6) gestational weeks; and Group 4, 24 women enrolled between 29 and 30 (+6) gestational weeks (n=24).

**Table 1:** Baseline characteristics of the intervention and control groups.

Variable		Total (N=269)	Control group (n=136)	Intervention group (n=133)	Wilcoxon rank sum or χ^2^ test	*P* value
Age (years), mean (SD)		31.07 (4.34)	30.93 (4.48)	31.23 (4.21)	0.95^a^	.34
**Age (years), n (%)**					0.04^b^	.84
	≤35	213 (79.1)	107 (78.7)	106 (79.7)		
	>35	56 (20.8)	29 (21.3)	27 (20.3)		
BMI, mean (SD)		24.04 (11.43)	25.07 (15.62)	22.99 (3.64)	–0.95^a^	.34
**BMI (kg/m^2^) , n (%)**					2.85^b^	.41
	10-18.5	24 (8.9)	12 (8.8)	12 (9.0)		
	18.5-24	137 (50.9)	69 (50.7)	68 (51.1)		
	24-28	71 (26.4)	32 (23.5)	39 (29.3)		
	≥28	37 (13.8)	23 (16.9)	14 (10.5)		
**Gravidity, n (%)**					0.04^b^	.84
	Primigravida	123 (45.7)	63 (46.3)	60 (45.1)		
	Multigravida	146 (54.3)	73 (53.7)	73 (54.9)		
**Parity, n (%)**					0.42^b^	.52
	Primipara	161 (59.9)	84 (61.8)	77 (57.9)		
	Multipara	108 (40.1)	52 (38.2)	56 (42.1)		
**Newborn sex, n (%)**					0.45^b^	.50
	Boy	134 (49.8)	65 (47.8)	69 (51.9)		
	Girl	135 (50.2)	71 (52.2)	64 (48.1)		
**Nationality, n (%)**					1.76^b^	.19
	Ethnic Han	259 (96.3)	133 (97.8)	126 (94.7)		
	Other	10 (3.7)	3 (2.2)	7 (5.3)		
**Family history of diabetes mellitus, n (%)**					1.30^b^	.25
	Yes	12 (4.5)	8 (5.9)	4 (3.0)		
	No	257 (95.5)	128 (94.1)	129 (97.0)		
**OGTT^c^values (mmol/L), mean (SD)**						
	Fasting	4.98 (0.78)	5.04 (0.75)	4.93 (0.81)	–1.56^a^	.12
	1 hour	10.20 (1.84)	10.03 (1.88)	10.37 (1.79)	0.87^a^	.39
	2 hour	8.89 (1.69)	8.83 (1.63)	8.96 (1.75)	–0.03^a^	.97
Gestational weeks at enrollment, mean (SD)		26.37 (1.74)	26.27 (1.72)	26.48 (1.77)	1.14^a^	.26
**Patient groups according to gestational weeks at enrollment, n (%)**					1.71^b^	.63
	Group 1	66 (24.5)	34 (25.0)	32 (24.1)		
	Group 2	113 (42.0)	61 (44.9)	52 (39.1)		
	Group 3	66 (24.5)	29 (21.3)	37 (27.8)		
	Group 4	24 (8.9)	12 (8.8)	12 (9.0)		

^a^Wilcoxon rank sum test.

^b^Chi-square test.

^c^OGTT: oral glucose tolerance test.

### Primary Outcome

The glycemic qualification rate of the intervention group was higher than that of the control group at nearly all time points in Groups 1 to 3, with a statistically significant difference at 3 time points: Group 1 at T3 (54.8% vs 83.3%) and Group 2 at T3 (62.5% vs 80.0%) and T7 (75.0% vs 100%) (Table 2).

**Table 2:** Glycemic qualification rate of the control group (CG) and intervention group (IG) according to gestational weeks at enrollment.

	Glycemic qualification rate (%), median (Q1, Q3)										
Time point	Group 1			Group 2			Group 3			Group 4	
	CG	IG	CG		IG	CG		IG	CG		IG
T1	59.17 (40.00, 80.00)	60.77 (40.00, 80.00)	60.00 (40.00, 80.00)		66.67 (41.67, 81.82)	50.00 (25.00, 61.90)		50.00 (50.00, 83.33)	48.33 (37.50, 73.33)		50.00 (33.33, 62.50)
T2	53.33 (40.00, 88.89)	75.00 (50.00, 100)	62.50 (37.50, 87.50)		70.00 (52.94, 90.00)	62.50 (40.00, 100)		61.25 (50.00, 100)	45.00 (30.00, 74.17)		62.50 (40.00, 100)
T3	54.79 (46.67, 80.00)	83.33 (62.50, 100)*	62.50 (50.00, 75.00)		80.00 (62.50, 87.50)*	62.50 (50.00, 90.00)		80.00 (50.00, 100)	52.78 (40.00, 87.50)		50.00 (26.67, 75.00)
T4	80.00 (50.00, 100)	100 (73.33, 100)	70.59 (60.00, 100)		80.00 (62.50, 100)	75.00 (53.33, 100)		75.00 (53.33, 100)	70.00 (T50.00, 100)		63.33 (57.14, 100)
T5	70.00 (50.00, 80.00)	87.50 (66.67, 100)	87.50 (62.50, 100)		80.00 (75.00, 100)	87.50 (62.50, 100)		87.50 (60.00, 100)	88.75 (73.75, 95.00)		62.50 (45.00, 82.50)
T6	87.50 (46.67, 100)	83.33 (57.35, 93.75)	75.00 (50.00, 100)		81.67 (75.00, 100)	75.00 (55.00, 100)		83.89 (50.00, 100)	75.00 (50.00, 80.00)		60.00 (60.00, 60.00)
T7	80.00 (70.00, 87.50)	88.89 (75.00, 100)	75.00 (60.00, 87.50)		100 (87.50, 100)*	75.00 (62.50, 81.25)		75.00 (60.00, 87.50)	–		–
T8	87.50 (40.00, 100)	100 (60.00, 100)	87.50 (60.00, 100)		90.97 (80.00, 100)	62.50 (62.50, 62.50)		100 (100, 100)	–		–
T9	50.00 (50.00, 50.00)	80.00 (80.00, 80.00)	75.00 (50.00, 100)		100 (100, 100)	–		–	–		–

*Statistically significant at *P*<.05.

With respect to intervention period, at T1, when participants began to receive health management, the BG qualification rates of the control and intervention groups were similar. At T2, the BG qualification rates in the control group and the intervention group both began to rise (53.3% vs 75.0%, respectively, in Group 1 and 62.5% vs 70.0%, respectively, in Group 2). At T3, when the intervention group had been managed for approximately 1 month, the BG qualification rates in the control and intervention groups were 54.8% vs 83.3%, respectively, in Group 1, 62.5% vs 80.0%, respectively, in Group 2, and 62.5% vs 80.0%, respectively, in Group 3.

With regard to gestational week of enrollment, the earlier the intervention started, the better BG was controlled. That is, in Group 1, who enrolled between 23 and 24 (+6) gestational weeks, the control rate was not different between the two group at T1, but once the intervention started, the BG control rate of the intervention group became much higher than that of the control group (75.0% vs 53.3%) at T2. The difference was seen until T5, and then decreased to less than 10% after that. In Group 2, who enrolled between 25 and 26 (+6) gestational weeks, the trend was similar, although the difference was only seen until T4 and then decreased. However, in Group 4, a difference in the BG control rate between the intervention and control groups was not observed.

**Figure 2:**
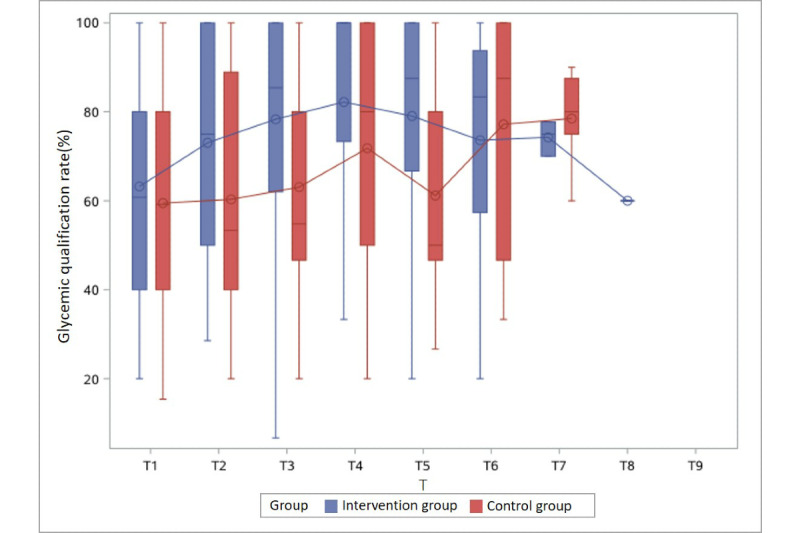
Glycemic qualification rate of the intervention and control groups in Group 1 (enrolled between 23 and 24 [+6] gestational weeks) at different time points. T: 2-week period.

**Figure 3:**
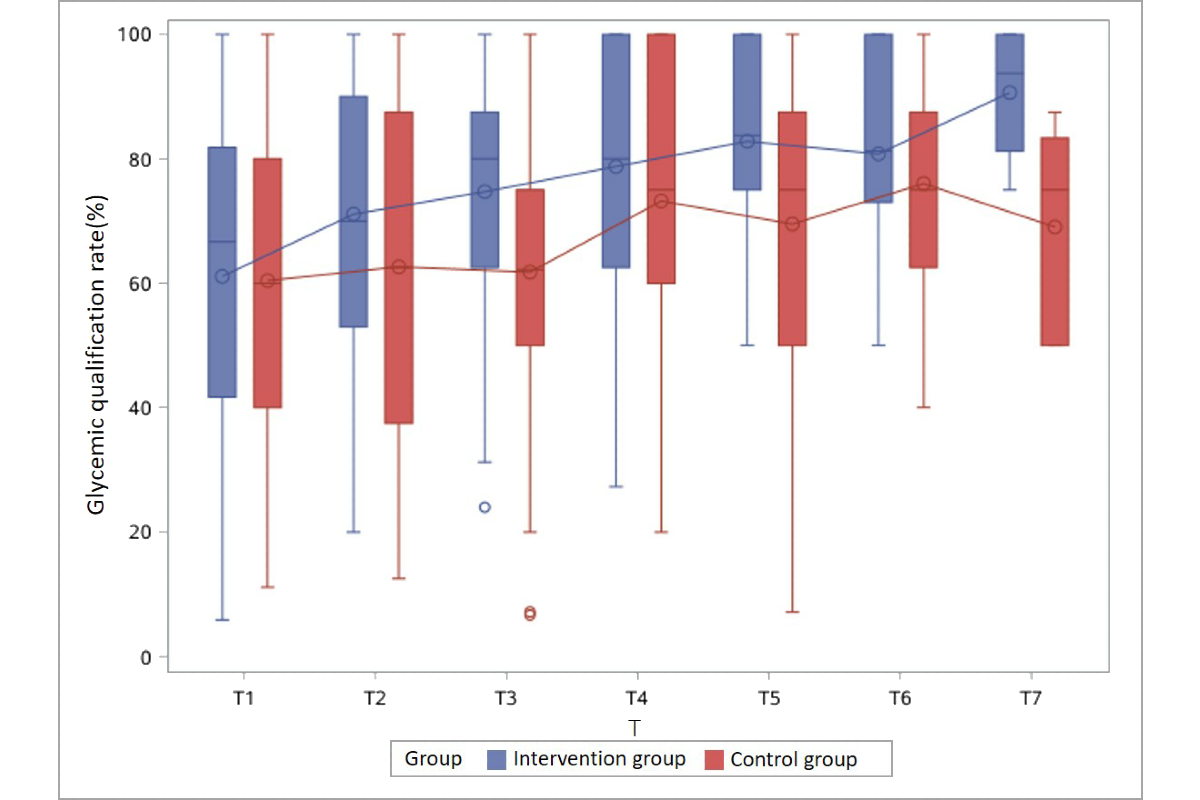
Glycemic qualification rate of the intervention and control groups in Group 2 (enrolled between 25 and 26 [+6] gestational weeks) at different time points. T: 2-week period.

**Figure 4:**
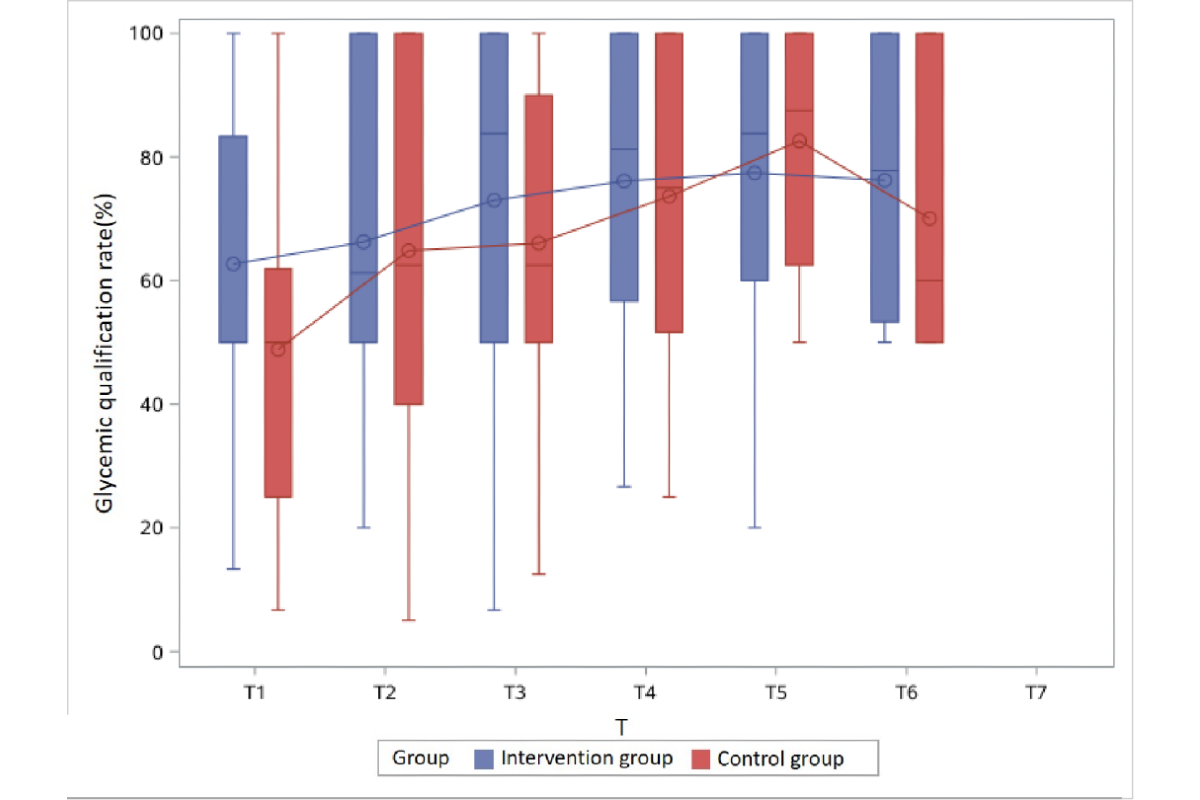
Glycemic qualification rate of the intervention and control groups in Group 3 (enrolled between 27 and 28 [+6] gestational weeks) at different time points. T: 2-week period.

**Figure 5:**
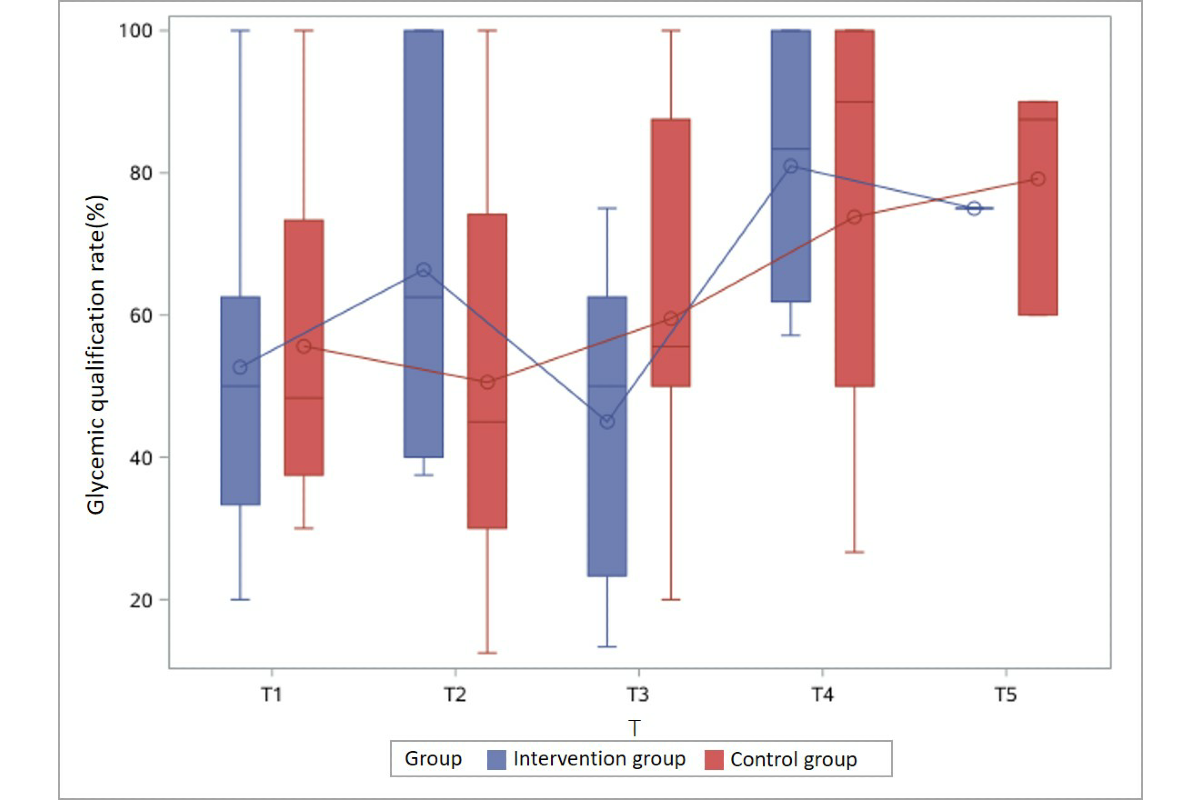
Glycemic qualification rate of the intervention and control groups in Group 4 (enrolled between 29 and 30 [+6] gestational weeks) at different time points. T: 2-week period.

Furthermore, the glycemic qualification rate gradually increased as gestational weeks progressed in both groups, regardless of the intervention or control group assignment (Table 2 and Figures 2-5).

### Secondary Outcomes

Secondary outcomes including delivery mode, premature rupture of the membranes, preterm birth, infant's birth weight, and postpartum hemorrhage were not significantly different between the control and intervention groups (see Multimedia Appendix 1, Tables 5-8).

## Discussion

### Principal Findings

This randomized controlled trial indicated that the use of instant messaging platforms such as WeChat in China for health education and lifestyle intervention by women with GDM for the entire GDM management period was associated with a higher rate of BG falling within the optimal range than with standard clinical prenatal care, although these results were not statistically significant. Clinical pregnancy outcomes were not significantly different between the intervention and control groups. Additionally, the increasing trend in the glycemic qualification rate was seemingly more stable in the WeChat management group than in the control group, which supports the nonnegligible role of continuous management. As well, GDM management was beneficial for improving the BG qualification rate in both groups regardless of when patients enrolled in the study, which means that when the intervention starts earlier, optimal BG is maintained longer and maternal benefits are more extensive. This highlights the importance of starting health management when GDM is diagnosed.

### Comparison with Previous Studies

Telemedicine, concentrating on the intervals between obstetric clinic visits, makes GDM management more comprehensive and complete in a limited time window. This can make pregnant women who are unconcerned or overwhelmed by guilt and anxiety feel more confident to manage their GDM. WeChat group chat provides a platform for effective education to ensure clear understanding of medical principles and to provide active pregnancy consultations instead of women having to wait for clinic visits every few weeks. This continuous BG and lifestyle management provides more individualized and adequate practical support than do doctors’ recommendations and medical guidelines alone and makes doctors’ orders more feasible in practice, which results in better adherence [18,26]. In other words, greater self-efficacy results in better patient management. In addition, among all kinds of patient education and lifestyle interventions, dietary changes are the most difficult recommendations to follow [14]. Factors that impede the implementation of medical recommendations include social activities such as eating out, cultural factors such as the consumption of specific kinds of food, family impacts such as the rejection of the concept of an individual serving size, obsolescent views on pregnancy, and women’s family-centered values and role commitments [27,28]. The WeChat group brings together a group of people with specific but similar needs and provides moral support and opportunities for peer communication and family education, making it easier to follow patient management principles. Furthermore, telemedicine has the potential to save time and money by reducing the number of hospital appointments for women with GDM and increase clinical pressure for doctors. This concept is underexplored.

Many studies have explored the effects of online health education and lifestyle management methods on women with GDM. This article is, to our knowledge, the first to interpret BG data from this perspective. Prior studies have indicated that online education and management can help improve BG control [20,29-31]. Our findings confirmed this, although the results of this trial were not statistically significant. The glycemic qualification rates remained higher at nearly all time points regardless of the gestational weeks when participants started receiving online GDM management. This trend became less obvious and stable as the enrollment time drew closer to delivery, which was especially evident in Group 4 and can be attributed to the decreased sample size of participants who had delivered in this group. Some of the previous studies showed no improvement in BG control but better patient satisfaction [18]. Regardless of the management methods, BG control improved during the entire period of pregnancy, as shown in our results and previous research [18]. However, a more detailed glycemic qualification rate revealed that trends did not always improve. We observed that the glycemic qualification rate in the control group did not always increase as it did in the intervention group in Groups 1 to 4. As studies have reported the harm in BG variability for both mothers and infants [32], this discovery emphasizes that continuous management may help decrease BG variability and result in better outcomes. Regarding pregnancy outcomes, a few studies showed relationships between telemedicine interventions and longer gestational periods and fewer preterm births [18], reduced odds of cesarean section and pregnancy-induced hypertension [33], higher Apgar scores and reduced neonatal hospitalization [29]. We failed to investigate these relationships. A common result was that it was difficult to demonstrate differences between groups in pregnancy outcomes [21,23,31]. The same is true for systematic reviews and meta-analyses [34,35]. Compared with previous work, we included an analysis of intervention duration. The glycemic qualification rate increased gradually after GDM management was initiated in both the intervention and control groups regardless of the enrollment time, although the rate in the WeChat group was always higher than that in the control group. It might be interpreted that the earlier that education and lifestyle interventions start, the less time the pregnant woman and fetus are exposed to hyperglycemia and the greater the likelihood of better outcomes.

### Strengths and Limitations

The strengths of this study are its multicenter randomized design with a relatively large sample size that allowed us to investigate the effect of both the use of online GDM management and the prolongation of intervention duration. We enrolled women diagnosed with GDM at different gestational weeks and divided them into 4 groups, allowing a more detailed interpretation of the effects of online management on GDM. Moreover, instant messaging platforms such as WeChat may have better generalization than specifically designed webpages or applications because they have fewer requirements for managed patients and require no additional operations except chatting and following the instructions of the managers of the group. In addition, the high frequency of communication in the chat group and the positive response to health management information indicated that this online management method was welcomed. We conducted brief interviews after the research and obtained positive responses about the intervention.

Limitations also exist in this study. We did not use a continuous glucose monitoring system (CGMS) to obtain BG data. There is a possibility that some glucose information was missed, but studies and guidelines consider that a CGMS is not necessary in women with noninsulin-dependent GDM [36]. Additionally, we failed to demonstrate differences in pregnancy outcomes, as did most previous research, but we could not conclude that the intervention did not have an impact on clinical outcomes. The number of glucose records decreased in the third trimester. We could not determine whether the subjects had monitored their BG but did not record it or whether they had not tested their BG, but the missing values had no effect on the results after assessment.

General management of women with noninsulin-dependent GDM requires lifestyle interventions to realize glycemic control and weight gain management. If we are to implement a more convenient intervention method, comprehensive assessment needs to be done—for example, by assessing its effects on weight change, BG control, sleep quality, and psychological status. An assessment of indicators of adherence is required to confirm the management effect. A larger sample size may help investigate the impact of telemedicine use on the pregnancy outcomes of women with GDM. This investigation is indispensable, as the aim of GDM management is to reduce negative outcomes of the pregnant woman and fetus through BG and weight control. Therefore, the lack of statistically significant results should be further explored in a larger population. A continuous management system for women with GDM to be implemented between prenatal clinics, allowing professional education to be more accessible, is considered to have the potential to reduce clinic visits and improve medical service quality. In the long run, this management method can be extended to GDM prevention at the beginning of pregnancy and GDM follow-up postpartum, emphasizing the continuity of healthy lifestyle education and intervention [37,38].

### Conclusions

This multicenter randomized controlled trial that enrolled noninsulin-dependent women with GDM demonstrated that using an instant messaging platform for health education and lifestyle intervention was more effective regarding BG control than standard clinical prenatal care. Further studies may investigate a more systematic method of GDM management conducted through online group chats. Differences in pregnancy outcomes were not significant. More exploration is needed to clarify this trend. These findings also call for more exploitation of telemedicine use for GDM prevention, patient management, and follow-up.

## Appendix

Multimedia Appendix 1 Baseline characteristics and pregnancy outcomes of each group.

Multimedia Appendix 2 CONSORT-eHEALTH checklist (V 1.6.1).

## Abbreviations

BG: blood glucose
CGMS: continuous glucose monitoring system
GDM: gestational diabetes mellitus
OGTT: oral glucose tolerance test

## References

[1] World Health Organization (2014). Diagnostic criteria and classification of hyperglycaemia first detected in pregnancy: a World Health Organization Guideline. Diabetes Res Clin Pract.

[2] National Institute for Health and Care Excellence (UK) (2015). Outcomes and risks for woman and baby. Diabetes in Pregnancy: Management of Diabetes and Its Complications from Preconception to the Postnatal Period.

[3] Metzger BE, Lowe LP, Dyer AR, Trimble ER, Chaovarindr U, Coustan DR, Hadden DR, McCance DR, Hod M, McIntyre HD, Oats JJN, Persson B, Rogers MS, Sacks DA, HAPO Study Cooperative Research Group (2008). Hyperglycemia and adverse pregnancy outcomes. N Engl J Med.

[4] Harris DL, Weston PJ, Signal M, Chase JG, Harding JE (2013). Dextrose gel for neonatal hypoglycaemia (the Sugar Babies Study): a randomised, double-blind, placebo-controlled trial. Lancet.

[5] Bellamy L, Casas J, Hingorani AD, Williams D (2009). Type 2 diabetes mellitus after gestational diabetes: a systematic review and meta-analysis. Lancet.

[6] Page KA, Romero A, Buchanan TA, Xiang AH (2014). Gestational diabetes mellitus, maternal obesity, and adiposity in offspring. J Pediatr.

[7] Ornoy A (2011). Prenatal origin of obesity and their complications: Gestational diabetes, maternal overweight and the paradoxical effects of fetal growth restriction and macrosomia. Reprod Toxicol.

[8] Dabelea D, Hanson RL, Lindsay RS, Pettitt DJ, Imperatore G, Gabir MM, Roumain J, Bennett PH, Knowler WC (2000). Intrauterine exposure to diabetes conveys risks for type 2 diabetes and obesity: a study of discordant sibships. Diabetes.

[9] Rosenberg TJ, Garbers S, Lipkind H, Chiasson MA (2005). Maternal obesity and diabetes as risk factors for adverse pregnancy outcomes: differences among 4 racial/ethnic groups. Am J Public Health.

[10] American Diabetes Association (2020). 14. Management of Diabetes in Pregnancy: Standards of Medical Care in Diabetes-2020. Diabetes Care.

[11] Martis R, Crowther CA, Shepherd E, Alsweiler J, Downie MR, Brown J (2018). Treatments for women with gestational diabetes mellitus: an overview of Cochrane systematic reviews. Cochrane Database Syst Rev.

[12] Nigam A, Sharma S, Varun N, Munjal YP, Prakash A (2019). Comparative analysis of 2-week glycaemic profile of healthy versus mild gestational diabetic pregnant women using flash glucose monitoring system: an observational study. BJOG.

[13] Cosson E, Baz B, Gary F, Pharisien I, Nguyen MT, Sandre-Banon D, Jaber Y, Cussac-Pillegand C, Banu I, Carbillon L, Valensi P (2017). Poor reliability and poor adherence to self-monitoring of blood glucose are common in women with gestational diabetes mellitus and may be associated with poor pregnancy outcomes. Diabetes Care.

[14] Sousa AMDS, Fiuza D, Mikami FCF, Abrão KC, Francisco RPV, Zugaib M (2016). Evaluation of information retention and adherence to treatment in patients with gestational diabetes mellitus after multidisciplinary group. Rev Assoc Med Bras (1992).

[15] Homko CJ, Deeb LC, Rohrbacher K, Mulla W, Mastrogiannis D, Gaughan J, Santamore WP, Bove AA (2012). Impact of a telemedicine system with automated reminders on outcomes in women with gestational diabetes mellitus. Diabetes Technol Ther.

[16] Hirst JE, Mackillop L, Loerup L, Kevat DA, Bartlett K, Gibson O, Kenworthy Y, Levy JC, Tarassenko L, Farmer A (2015). Acceptability and user satisfaction of a smartphone-based, interactive blood glucose management system in women with gestational diabetes mellitus. J Diabetes Sci Technol.

[17] Given JE, Bunting BP, O'Kane MJ, Dunne F, Coates VE (2015). Tele-Mum: A Feasibility Study for a Randomized Controlled Trial Exploring the Potential for Telemedicine in the Diabetes Care of Those with Gestational Diabetes. Diabetes Technol Ther.

[18] Mackillop L, Hirst JE, Bartlett KJ, Birks JS, Clifton L, Farmer AJ, Gibson O, Kenworthy Y, Levy JC, Loerup L, Rivero-Arias O, Ming W, Velardo C, Tarassenko L (2018). Comparing the efficacy of a mobile phone-based blood glucose management system with standard clinic care in women with gestational diabetes: Randomized controlled trial. JMIR Mhealth Uhealth.

[19] Alqudah A, McMullan P, Todd A, O'Doherty C, McVey A, McConnell M, O'Donoghue J, Gallagher J, Watson CJ, McClements L (2019). Service evaluation of diabetes management during pregnancy in a regional maternity hospital: potential scope for increased self-management and remote patient monitoring through mHealth solutions. BMC Health Serv Res.

[20] Al-Ofi EA, Mosli HH, Ghamri KA, Ghazali SM (2019). Management of postprandial hyperglycaemia and weight gain in women with gestational diabetes mellitus using a novel telemonitoring system. J Int Med Res.

[21] Homko CJ, Santamore WP, Whiteman V, Bower M, Berger P, Geifman-Holtzman O, Bove AA (2007). Use of an internet-based telemedicine system to manage underserved women with gestational diabetes mellitus. Diabetes Technol Ther.

[22] Pérez-Ferre N, Galindo M, Fernández MD, Velasco V, Runkle I, de la Cruz MJ, Martín Rojas-Marcos P, Del Valle L, Calle-Pascual AL (2010). The outcomes of gestational diabetes mellitus after a telecare approach are not inferior to traditional outpatient clinic visits. Int J Endocrinol.

[23] Carral F, Ayala MDC, Fernández JJ, González C, Piñero A, García G, Cañavate C, Jiménez AI, García C (2015). Web-based telemedicine system is useful for monitoring glucose control in pregnant women with diabetes. Diabetes Technol Ther.

[24] Metzger BE, Gabbe SG, Persson B, Buchanan TA, Catalano PA, Damm P, Dyer AR, Leiva AD, Hod M, Kitzmiler JL, Lowe LP, McIntyre HD, Oats JJN, Omori Y, Schmidt MI, International Association of Diabetes Pregnancy Study Groups Consensus Panel (2010). International association of diabetes and pregnancy study groups recommendations on the diagnosis and classification of hyperglycemia in pregnancy. Diabetes Care.

[25] American Diabetes Association (2019). 14. Management of Diabetes in Pregnancy: Standards of Medical Care in Diabetes-2019. Diabetes Care.

[26] Martis R, Brown J, Crowther CA (2017). Views and Experiences of New Zealand Women with Gestational Diabetes in Achieving Glycaemic Control Targets: The Views Study. J Diabetes Res.

[27] Wah YYE, McGill M, Wong J, Ross GP, Harding A, Krass I (2019). Self-management of gestational diabetes among Chinese migrants: A qualitative study. Women Birth.

[28] Dennison RA, Ward RJ, Griffin SJ, Usher-Smith JA (2019). Women's views on lifestyle changes to reduce the risk of developing Type 2 diabetes after gestational diabetes: a systematic review, qualitative synthesis and recommendations for practice. Diabet Med.

[29] Kolivand M, Rahimi MA, Keramat A, Shariati M, Emamian MH (2019). Effect of a new self-care guide package on maternal and neonatal outcomes in gestational diabetes: A randomized control trial. J Diabetes.

[30] Guo H, Zhang Y, Li P, Zhou P, Chen L, Li S (2019). Evaluating the effects of mobile health intervention on weight management, glycemic control and pregnancy outcomes in patients with gestational diabetes mellitus. J Endocrinol Invest.

[31] Rasekaba TM, Furler J, Young D, Liew D, Gray K, Blackberry I, Lim WK (2018). Using technology to support care in gestational diabetes mellitus: Quantitative outcomes of an exploratory randomised control trial of adjunct telemedicine for gestational diabetes mellitus (TeleGDM). Diabetes Res Clin Pract.

[32] Suh S, Kim JH (2015). Glycemic variability: How do we measure it and why is it important?. Diabetes Metab J.

[33] Yang P, Lo W, He Z, Xiao X (2018). Medical nutrition treatment of women with gestational diabetes mellitus by a telemedicine system based on smartphones. J Obstet Gynaecol Res.

[34] Ming W, Mackillop LH, Farmer AJ, Loerup L, Bartlett K, Levy JC, Tarassenko L, Velardo C, Kenworthy Y, Hirst JE (2016). Telemedicine technologies for diabetes in pregnancy: A systematic review and meta-analysis. J Med Internet Res.

[35] Lau Y, Htun TP, Wong SN, Tam WSW, Klainin-Yobas P (2016). Efficacy of internet-based self-monitoring interventions on maternal and neonatal outcomes in perinatal diabetic women: A systematic review and meta-analysis. J Med Internet Res.

[36] Raman P, Shepherd E, Dowswell T, Middleton P, Crowther CA (2017). Different methods and settings for glucose monitoring for gestational diabetes during pregnancy. Cochrane Database Syst Rev.

[37] Zilberman-Kravits D, Meyerstein N, Abu-Rabia Y, Wiznitzer A, Harman-Boehm I (2018). The impact of a cultural lifestyle intervention on metabolic parameters after gestational diabetes mellitus: a randomized controlled trial. Matern Child Health J.

[38] Koivusalo SB, Rönö Kristiina, Klemetti MM, Roine RP, Lindström Jaana, Erkkola M, Kaaja RJ, Pöyhönen-Alho Maritta, Tiitinen A, Huvinen E, Andersson S, Laivuori H, Valkama A, Meinilä Jelena, Kautiainen H, Eriksson JG, Stach-Lempinen B (2016). Gestational Diabetes Mellitus Can Be Prevented by Lifestyle Intervention: The Finnish Gestational Diabetes Prevention Study (RADIEL): A Randomized Controlled Trial. Diabetes Care.

