# Supplementary material for: Comparing the Efficacies of Telemedicine and Standard Prenatal Care on Blood Glucose Control in Women With Gestational Diabetes Mellitus: Randomized Controlled Trial
Source: JMIR Mhealth Uhealth. 2021 May 25;9(5):e22881. doi: 10.2196/22881 (PMC8188321; doi:10.2196/22881)
Supplement: Multimedia Appendix 1 [file mhealth_v9i5e22881_app1.docx]

Multimedia Appendix table 1. Baseline characteristics of participants enrolled between 23 and 24^+6^ gestational weeks in the intervention and control group.

| Variable | Group Ⅰ | | | | |  | *P* |
| --- | --- | --- | --- | --- | --- | --- | --- |
|  | Total (N=67) |  | Control group (N=35) |  | Intervention group (N=32) |  |  |
| Age (years) |  |  |  |  |  |  |  |
| Mean (SD) | 30.54 (3.94) |  | 30.34 (4.18) |  | 30.75 (3.72) | -0.42^a^ | .68 |
| Age (years) |  |  |  |  |  |  |  |
| ≤35 (%) | 57 (85.07) |  | 30 (85.71) |  | 27 (84.38) | 0.02^b^ | .88 |
| >35 (%) | 10 (14.93) |  | 5 (14.29) |  | 5 (15.63) |  |  |
| BMI |  |  |  |  |  |  |  |
| Mean (SD) | 24.27 (5.12) |  | 24.63 (6.30) |  | 23.87 (3.45) | 0.19^c^ | .85 |
| BMI group |  |  |  |  |  |  |  |
| 10-18.5(%) | 4 (5.97) |  | 3 (8.57) |  | 1 (3.13) | 4.15^b^ | .25 |
| 18.5-24(%) | 31 (46.27) |  | 16 (45.71) |  | 15 (46.88) |  |  |
| 24-28(%) | 19 (28.36) |  | 7 (20.00) |  | 12 (37.50) |  |  |
| ≥28(%) | 13 (19.40) |  | 9 (25.71) |  | 4 (12.50) |  |  |
| Gravidity |  |  |  |  |  |  |  |
| First pregnancy (%) | 33 (49.25) |  | 18 (51.43) |  | 15 (46.88) | 0.14^b^ | .71 |
| Nonfirst pregnancy (%) | 34 (50.75) |  | 17 (48.57) |  | 17 (53.13) |  |  |
| Parity |  |  |  |  |  |  |  |
| Primipara (%) | 45 (67.16) |  | 24 (68.57) |  | 21 (65.63) | 0.07^b^ | .80 |
| Multipara (%) | 22 (32.84) |  | 11 (31.43) |  | 11 (34.38) |  |  |
| Newborn sex |  |  |  |  |  |  |  |
| Boy (%) | 33 (49.25) |  | 15 (42.86) |  | 18 (56.25) | 1.20^b^ | .27 |
| Girl (%) | 34 (50.75) |  | 20 (57.14) |  | 14 (43.75) |  |  |
| Nationality |  |  |  |  |  |  |  |
| Ethnic Han (%) | 62 (92.54) |  | 34 (97.14) |  | 28 (87.50) | 2.25^b^ | .13 |
| Others (%) | 5 (7.46) |  | 1 (2.86) |  | 4 (12.50) |  |  |
| Family history of diabetes mellitus |  |  |  |  |  |  |  |
| Yes (%) | 1 (1.49) |  | 1 (2.86) |  | 0 (0.00) | 0.93^b^ | .34 |
| No (%) | 66 (98.51) |  | 34 (97.14) |  | 32 (100.0) |  |  |
| Family history of hypertension |  |  |  |  |  |  |  |
| Yes (%) | 5 (7.46) |  | 3 (8.57) |  | 2 (6.25) | 0.13^b^ | .72 |
| No (%) | 62 (92.54) |  | 32 (91.43) |  | 30 (93.75) |  |  |
| Enrollment gestational weeks |  |  |  |  |  |  |  |
| Mean (SD) | 24.37 (0.40) |  | 24.40 (0.37) |  | 24.34 (0.44) | -0.27^c^ | .79 |
| OGTT-fasting |  |  |  |  |  |  |  |
| Mean (SD) | 5.00 (0.74) |  | 5.02 (0.68) |  | 4.98 (0.81) | -0.56^c^ | .57 |
| OGTT-1 hour |  |  |  |  |  |  |  |
| Mean (SD) | 9.80 (1.92) |  | 9.66 (2.18) |  | 9.95 (1.62) | -0.61^a^ | .54 |
| OGTT-2 hour |  |  |  |  |  |  |  |
| Mean (SD) | 8.74 (1.42) |  | 8.67 (1.54) |  | 8.81 (1.31) | -0.4^a^ | .69 |

^a^ Independent-samples T test

^b^ **Chi-squared test**

^c^ **Wilcoxon rank sum test**

Multimedia Appendix table 2. Baseline characteristics of participants enrolled between 25 and 26^+6^ gestational weeks in the intervention and control group.

| Variable | Group Ⅱ | | | | |  | *P* |
| --- | --- | --- | --- | --- | --- | --- | --- |
|  | Total (N=113) |  | Control group (N=61) |  | Intervention group (N=52) |  |  |
| Age (years) |  |  |  |  |  |  |  |
| Mean (SD) | 31.09 (4.18) |  | 30.57 (4.38) |  | 31.69 (3.88) | -1.42^a^ | .16 |
| Age (years) |  |  |  |  |  |  |  |
| ≤35 (%) | 90 (79.65) |  | 49 (80.33) |  | 41 (78.85) | 0.04^b^ | .85 |
| >35 (%) | 23 (20.35) |  | 12 (19.67) |  | 11 (21.15) |  |  |
| BMI |  |  |  |  |  |  |  |
| Mean (SD) | 24.47 (16.82) |  | 25.78 (22.69) |  | 22.94 (3.42) | 0.21^c^ | .84 |
| BMI group |  |  |  |  |  |  |  |
| 10-18.5(%) | 10 (8.85) |  | 7 (11.48) |  | 3 (5.77) | 4.44^b^ | .22 |
| 18.5-24(%) | 63 (55.75) |  | 34 (55.74) |  | 29 (55.77) |  |  |
| 24-28(%) | 29 (25.66) |  | 12 (19.67) |  | 17 (32.69) |  |  |
| ≥28(%) | 11 (9.73) |  | 8 (13.11) |  | 3 (5.77) |  |  |
| Gravidity |  |  |  |  |  |  |  |
| First pregnancy (%) | 49 (43.36) |  | 28 (45.90) |  | 21 (40.38) | 0.35^b^ | .56 |
| Nonfirst pregnancy (%) | 64 (56.64) |  | 33 (54.10) |  | 31 (59.62) |  |  |
| Parity |  |  |  |  |  |  |  |
| Primipara (%) | 65 (57.52) |  | 39 (63.93) |  | 26 (50.00) | 2.23^b^ | .14 |
| Multipara (%) | 48 (42.48) |  | 22 (36.07) |  | 26 (50.00) |  |  |
| Newborn sex |  |  |  |  |  |  |  |
| Boy (%) | 56 (49.56) |  | 31 (50.82) |  | 25 (48.08) | 0.08^b^ | .77 |
| Girl (%) | 57 (50.44) |  | 30 (49.18) |  | 27 (51.92) |  |  |
| Nationality |  |  |  |  |  |  |  |
| Ethnic Han (%) | 109 (96.46) |  | 59 (96.72) |  | 50 (96.15) | 0.03^b^ | .87 |
| Others (%) | 4 (3.54) |  | 2 (3.28) |  | 2 (3.85) |  |  |
| Family history of diabetes mellitus |  |  |  |  |  |  |  |
| Yes (%) | 5 (4.42) |  | 4 (6.56) |  | 1 (1.92) | 1.43^b^ | .23 |
| No (%) | 108 (95.58) |  | 57 (93.44) |  | 51 (98.08) |  |  |
| Family history of hypertension |  |  |  |  |  |  |  |
| Yes (%) | 2 (1.77) |  | 1 (1.64) |  | 1 (1.92) | 0.01^b^ | .91 |
| No (%) | 111 (98.23) |  | 60 (98.36) |  | 51 (98.08) |  |  |
| Enrollment gestational weeks |  |  |  |  |  |  |  |
| Mean (SD) | 25.87 (0.54) |  | 25.78 (0.54) |  | 25.98 (0.53) | 1.98^c^ | .048 |
| OGTT-fasting |  |  |  |  |  |  |  |
| Mean (SD) | 4.90 (0.68) |  | 4.91 (0.76) |  | 4.88 (0.57) | 0.24^c^ | .81 |
| OGTT-1 hour |  |  |  |  |  |  |  |
| Mean (SD) | 10.01 (1.53) |  | 9.79 (1.51) |  | 10.27 (1.53) | 1.44^c^ | .15 |
| OGTT-2 hour |  |  |  |  |  |  |  |
| Mean (SD) | 8.70 (1.50) |  | 8.60 (1.49) |  | 8.83 (1.51) | -0.79^a^ | .43 |

^a^ Independent-samples T test

^b^ **Chi-squared test**

^c^ **Wilcoxon rank sum test**

Multimedia Appendix table 3. Baseline characteristics of participants enrolled between 27 and 28^+6^ gestational weeks in the intervention and control group.

| Variable | Group Ⅲ | | | | |  | *P* |
| --- | --- | --- | --- | --- | --- | --- | --- |
|  | Total (N=67) |  | Control group (N=26) |  | Intervention group (N=38) |  |  |
| Age (years) |  |  |  |  |  |  |  |
| Mean (SD) | 30.85 (4.77) |  | 31.07 (4.63) |  | 30.68 (4.93) | 0.53^c^ | .60 |
| Age (years) |  |  |  |  |  |  |  |
| ≤35 (%) | 54 (80.60) |  | 23 (79.31) |  | 31 (81.58) | 0.05^b^ | .82 |
| >35 (%) | 13 (19.40) |  | 6 (20.69) |  | 7 (18.42) |  |  |
| BMI |  |  |  |  |  |  |  |
| Mean (SD) | 23.17 (4.26) |  | 24.24 (4.25) |  | 22.35 (4.14) | 1.83^a^ | .07 |
| BMI group |  |  |  |  |  |  |  |
| 10-18.5(%) | 10 (14.93) |  | 2 (6.90) |  | 8 (21.05) | 2.90^b^ | .41 |
| 18.5-24(%) | 31 (46.27) |  | 14 (48.28) |  | 17 (44.74) |  |  |
| 24-28(%) | 17 (25.37) |  | 9 (31.03) |  | 8 (21.05) |  |  |
| ≥28(%) | 9 (13.43) |  | 4 (13.79) |  | 5 (13.16) |  |  |
| Gravidity |  |  |  |  |  |  |  |
| First pregnancy (%) | 34 (50.75) |  | 13 (44.83) |  | 21 (55.26) | 0.72^b^ | .40 |
| Nonfirst pregnancy (%) | 33 (49.25) |  | 16 (55.17) |  | 17 (44.74) |  |  |
| Parity |  |  |  |  |  |  |  |
| Primipara (%) | 42 (62.69) |  | 17 (58.62) |  | 25 (65.79) | 0.36^b^ | .55 |
| Multipara (%) | 25 (37.31) |  | 12 (41.38) |  | 13 (34.21) |  |  |
| Newborn sex |  |  |  |  |  |  |  |
| Boy (%) | 32 (47.76) |  | 11 (37.93) |  | 21 (55.26) | 1.98^b^ | .16 |
| Girl (%) | 35 (52.24) |  | 18 (62.07) |  | 17 (44.74) |  |  |
| Nationality |  |  |  |  |  |  |  |
| Ethnic Han (%) | 67 (100.0) |  | 29 (100.0) |  | 38 (100.0) | - | - |
| Others (%) | 0 (0) |  | 0 (0) |  | 0 (0) |  |  |
| Family history of diabetes mellitus |  |  |  |  |  |  |  |
| Yes (%) | 5 (7.46) |  | 2 (6.90) |  | 3 (7.89) | 0.02^b^ | .88 |
| No (%) | 62 (92.54) |  | 27 (93.10) |  | 35 (92.11) |  |  |
| Family history of hypertension |  |  |  |  |  |  |  |
| Yes (%) | 5 (7.46) |  | 2 (6.90) |  | 3 (7.89) | 0.02^b^ | .88 |
| No (%) | 62 (92.54) |  | 27 (93.10) |  | 35 (92.11) |  |  |
| Enrollment gestational weeks |  |  |  |  |  |  |  |
| Mean (SD) | 27.97 (0.55) |  | 28.02 (0.55) |  | 27.92 (0.55) | 0.74^a^ | .46 |
| OGTT-fasting |  |  |  |  |  |  |  |
| Mean (SD) | 5.15 (0.98) |  | 5.35 (0.78) |  | 5.00 (1.09) ^*^ | 2.37^c^ | .018 |
| OGTT-1 hour |  |  |  |  |  |  |  |
| Mean (SD) | 10.90 (2.13) |  | 10.87 (1.97) |  | 10.93 (2.27) | 0.50^c^ | .62 |
| OGTT-2 hour |  |  |  |  |  |  |  |
| Mean (SD) | 9.36 (2.07) |  | 9.31 (1.82) |  | 9.41 (2.26) | 0.60^c^ | .55 |

^a^ Independent-samples T test

^b^ **Chi-squared test**

^c^ **Wilcoxon rank sum test**

Multimedia Appendix table 4. Baseline characteristics of participants enrolled between 29 and 30^+6^ gestational weeks in the intervention and control group.

| Variable | Group Ⅳ | | | | |  | *P* |
| --- | --- | --- | --- | --- | --- | --- | --- |
|  | Total (N=24) |  | Control group (N=12) |  | Intervention group (N=12) |  |  |
| Age (years) |  |  |  |  |  |  |  |
| Mean (SD) | 33.71 (4.70) |  | 34.33 (4.38) |  | 33.08 (5.12) | 0.64^a^ | .53 |
| Age (years) |  |  |  |  |  |  |  |
| ≤35 (%) | 13 (54.17) |  | 6 (50.00) |  | 7 (58.33) | 0.17^b^ | .68 |
| >35 (%) | 11 (45.83) |  | 6 (50.00) |  | 5 (41.67) |  |  |
| BMI |  |  |  |  |  |  |  |
| Mean (SD) | 23.59 (3.59) |  | 24.25 (4.02) |  | 22.93 (3.14) | 0.89^a^ | .38 |
| BMI group |  |  |  |  |  |  |  |
| 10-18.5(%) | 0 (0) |  | 0 (0) |  | 0 (0) | 0.95^b^ | .62 |
| 18.5-24(%) | 14 (58.33) |  | 6 (50.00) |  | 8 (66.67) |  |  |
| 24-28(%) | 6 (25.00) |  | 4 (33.33) |  | 2 (16.67) |  |  |
| ≥28(%) | 4 (16.67) |  | 2 (16.67) |  | 2 (16.67) |  |  |
| Gravidity |  |  |  |  |  |  |  |
| First pregnancy (%) | 7 (29.17) |  | 4 (33.33) |  | 3 (25.00) | 0.20^b^ | .65 |
| Nonfirst pregnancy (%) | 17 (70.83) |  | 8 (66.67) |  | 9 (75.00) |  |  |
| Parity |  |  |  |  |  |  |  |
| Primipara (%) | 9 (37.50) |  | 4 (33.33) |  | 5 (41.67) | 0.18^b^ | .67 |
| Multipara (%) | 15 (62.50) |  | 8 (66.67) |  | 7 (58.33) |  |  |
| Newborn sex |  |  |  |  |  |  |  |
| Boy (%) | 14 (58.33) |  | 8 (66.67) |  | 6 (50.00) | 0.69^b^ | .41 |
| Girl (%) | 10 (41.67) |  | 4 (33.33) |  | 6 (50.00) |  |  |
| Nationality |  |  |  |  |  |  |  |
| Ethnic Han (%) | 23 (95.83) |  | 12 (100.0) |  | 11 (91.67) | 1.04^b^ | .31 |
| Others (%) | 1 (4.17) |  | 0 (0.00) |  | 1 (8.33) |  |  |
| Family history of diabetes mellitus |  |  |  |  |  |  |  |
| Yes (%) | 1 (4.17) |  | 1 (8.33) |  | 0 (0.00) | 1.04^b^ | .31 |
| No (%) | 23 (95.83) |  | 11 (91.67) |  | 12 (100.0) |  |  |
| Family history of hypertension |  |  |  |  |  |  |  |
| Yes (%) | 0 (0) |  | 0 (0) |  | 0 (0) | - | - |
| No (%) | 24 (100.0) |  | 12 (100.0) |  | 12 (100.0) |  |  |
| Enrollment gestational weeks |  |  |  |  |  |  |  |
| Mean (SD) | 29.82 (0.59) |  | 29.74 (0.56) |  | 29.90 (0.64) | -0.68^a^ | .50 |
| OGTT-fasting |  |  |  |  |  |  |  |
| Mean (SD) | 4.89 (0.70) |  | 4.87 (0.68) |  | 4.90 (0.75) | -0.10^a^ | .92 |
| OGTT-1 hour |  |  |  |  |  |  |  |
| Mean (SD) | 10.41 (1.72) |  | 10.33 (1.94) |  | 10.50 (1.55) | -0.24^a^ | .82 |
| OGTT-2 hour |  |  |  |  |  |  |  |
| Mean (SD) | 9.02 (1.88) |  | 9.40 (1.86) |  | 8.64 (1.90) | 0.99^a^ | .33 |

^a^ Independent-samples T test

^b^ **Chi-squared test**

Multimedia Appendix table 5. Pregnancy outcomes of participants enrolled between 23 and 24^+6^ gestational weeks in the intervention group and control group

| Variable | Group Ⅰ | | | | X^2^ | | *P* |
| --- | --- | --- | --- | --- | --- | --- | --- |
|  | Total (N=66) | Control group (N=34) | Intervention group (N=32) | |  |  |  |
| Neonatal birth weight | |  |  | |  | |  |
| Normal (%) | 53 (80.30) | 28 (82.35) | 25 (78.13) | | 0.84^a^ | | .66 |
| Low birth weight (%) | 3 (4.55) | 2 (5.88) | 1 (3.13) | |  | |  |
| Macrosomia (%) | 10 (15.15) | 4 (11.76) | 6 (18.75) | |  | |  |
| Time of delivery | |  |  | |  | |  |
| Full term birth (%) | 62 (93.94) | 31 (91.18) | 31 (96.88) | | 0.94^a^ | | .33 |
| Preterm birth (%) | 4 (6.06) | 3 (8.82) | 1 (3.13) | |  | |  |
| Delivery mode | |  |  | |  | |  |
| Cesarean section (%) | 34 (51.52) | 16 (47.06) | 18 (56.25) | | 0.56^a^ | | .46 |
| Vaginally delivery (%) | 32 (48.48) | 18 (52.94) | 14 (43.75) | | |  | |
| Neonatal birth weight | |  |  | |  | |  |
| Yes (%) | 7 (10.61) | 2 (5.88) | 5 (15.63) | | 1.65^a^ | | .20 |
| No (%) | 59 (89.39) | 32 (94.12) | 27 (84.38) | | |  | |
| Postpartum hemorrhage | |  |  | |  | |  |
| Yes (%) | 2 (6.06) | 1 (6.25) | 1 (5.88) | | 0.00^a^ | | .96 |
| No (%) | 31 (93.94) | 15 (93.75) | 16 (94.12) |  | | | |

^a^ **Chi-squared test**

Multimedia Appendix table 6. Pregnancy outcomes of participants enrolled between 25 and 26^+6^ gestational weeks in the intervention group and control group

| Variable | Group II | | | X^2^ | | *P* |
| --- | --- | --- | --- | --- | --- | --- |
|  | Total (N=113) | Control group (N=61) | Intervention group (N=52) |  |  |  |
| Neonatal birth weight | |  |  |  | |  |
| Normal (%) | 100(88.50) | 54 (88.52) | 46 (88.46) | 1.94^a^ | | .38 |
| Low birth weight (%) | 4 (3.54) | 1 (1.64) | 3 (5.77) |  | |  |
| Macrosomia (%) | 9 (7.96) | 6 (9.84) | 3 (5.77) |  | |  |
| Time of delivery | |  |  |  | |  |
| Full term birth (%) | 108(95.58) | 59 (96.72) | 49 (94.23) | 0.41^a^ | | .52 |
| Preterm birth (%) | 5 (4.42) | 2 (3.28) | 3 (5.77) |  | |  |
| Delivery mode | |  |  |  | |  |
| Cesarean section (%) | 39 (34.51) | 18 (29.51) | 21 (40.38) | 1.47^a^ | | .23 |
| Vaginally delivery (%) | 74 (65.49) | 43 (70.49) | 31 (59.62) |  | | |
| Neonatal birth weight | |  |  |  | |  |
| Yes (%) | 17 (15.04) | 10 (16.39) | 7 (13.46) | 0.19^a^ | | .66 |
| No (%) | 96 (84.96) | 51 (83.61) | 45 (86.54) | | |  |
| Postpartum hemorrhage | |  |  |  | |  |
| Yes (%) | 0 | 0 | 0 | —— | | —— |
| No (%) | 36 (100.0) | 18 (100.0) | 18 (100.0) | |  | |

^a^ **Chi-squared test**

Multimedia Appendix table 7. Pregnancy outcomes of participants enrolled between 27 and 28^+6^ gestational weeks in the intervention group and control group

| Variable | Group III | | | X^2^ | | *P* |
| --- | --- | --- | --- | --- | --- | --- |
|  | Total (N=66) | Control group (N=29) | Intervention group (N=37) |  |  |  |
| Neonatal birth weight | |  |  |  | |  |
| Normal (%) | 59 (89.39) | 26 (89.66) | 33 (89.19) | 3.92^a^ | | .14 |
| Low birth weight (%) | 3 (4.55) | 0 (0.00) | 3 (8.11) |  | |  |
| Macrosomia (%) | 4 (6.06) | 3 (10.34) | 1 (2.70) |  | |  |
| Time of delivery | |  |  |  | |  |
| Full term birth (%) | 64 (96.97) | 29 (100.0) | 35 (94.59) | 1.62^a^ | | .20 |
| Preterm birth (%) | 2 (3.03) | 0 (0.00) | 2 (5.41) |  | |  |
| Delivery mode | |  |  |  | |  |
| Cesarean section (%) | 26 (39.39) | 12 (41.38) | 14 (37.84) | 0.09^a^ | | .77 |
| Vaginally delivery (%) | 40 (60.61) | 17 (58.62) | 23 (62.16) |  | | |
| Neonatal birth weight | |  |  |  | |  |
| Yes (%) | 6 (9.09) | 3 (10.34) | 3 (8.11) | 0.10^a^ | | .75 |
| No (%) | 60 (90.91) | 26 (89.66) | 34 (91.89) |  | | |
| Postpartum hemorrhage | |  |  |  | |  |
| Yes (%) | 0 | 0 | 0 | —— | | —— |
| No (%) | 25 (100.0) | 12 (100.0) | 13 (100.0) | |  | |

^a^ **Chi-squared test**

Multimedia Appendix table 8. Pregnancy outcomes of participants enrolled between 29 and 30^+6^ gestational weeks in the intervention group and control group

| Variable | Group IV | | | X^2^ | *P* |
| --- | --- | --- | --- | --- | --- |
|  | Total (N=24) | Control group (N=12) | Intervention group (N=12) |  |  |
| Neonatal birth weight | |  |  |  |  |
| Normal (%) | 20 (83.33) | 11 (91.67) | 9 (75.00) | 2.20^a^ | .33 |
| Low birth weight (%) | 2 (8.33) | 0 (0.00) | 2 (16.67) |  |  |
| Macrosomia (%) | 2 (8.33) | 1 (8.33) | 1 (8.33) |  |  |
| Time of delivery | |  |  |  |  |
| Full term birth (%) | 21 (87.50) | 12 (100.0) | 9 (75.00) | 3.43^a^ | .06 |
| Preterm birth (%) | 3 (12.50) | 0 (0.00) | 3 (25.00) |  |  |
| Delivery mode | |  |  |  |  |
| Cesarean section (%) | 13 (54.17) | 6 (50.00) | 7 (58.33) | 0.17^a^ | .68 |
| Vaginally delivery (%) | 11 (45.83) | 6 (50.00) | 5 (41.67) |  |  |
| Neonatal birth weight | |  |  |  |  |
| Yes (%) | 3 (12.50) | 2 (16.67) | 1 (8.33) | 0.38^a^ | .54 |
| No (%) | 21 (87.50) | 10 (83.33) | 11 (91.67) |  | |
| Postpartum hemorrhage | |  |  |  |  |
| Yes (%) | 1 (7.69) | 1 (16.67) | 0 (0.00) | 1.26^a^ | .26 |
| No (%) | 12 (92.31) | 5 (83.33) | 7 (100.0) |  |  |

^a^ **Chi-squared test**
